# Supplementary figures and images for: Characterization of the populations of upside-down jellyfish in Jardines de la Reina National Park, Cuba
Source: PeerJ. 2023 Apr 25;11:e15254. doi: 10.7717/peerj.15254 (PMC10143600; doi:10.7717/peerj.15254)

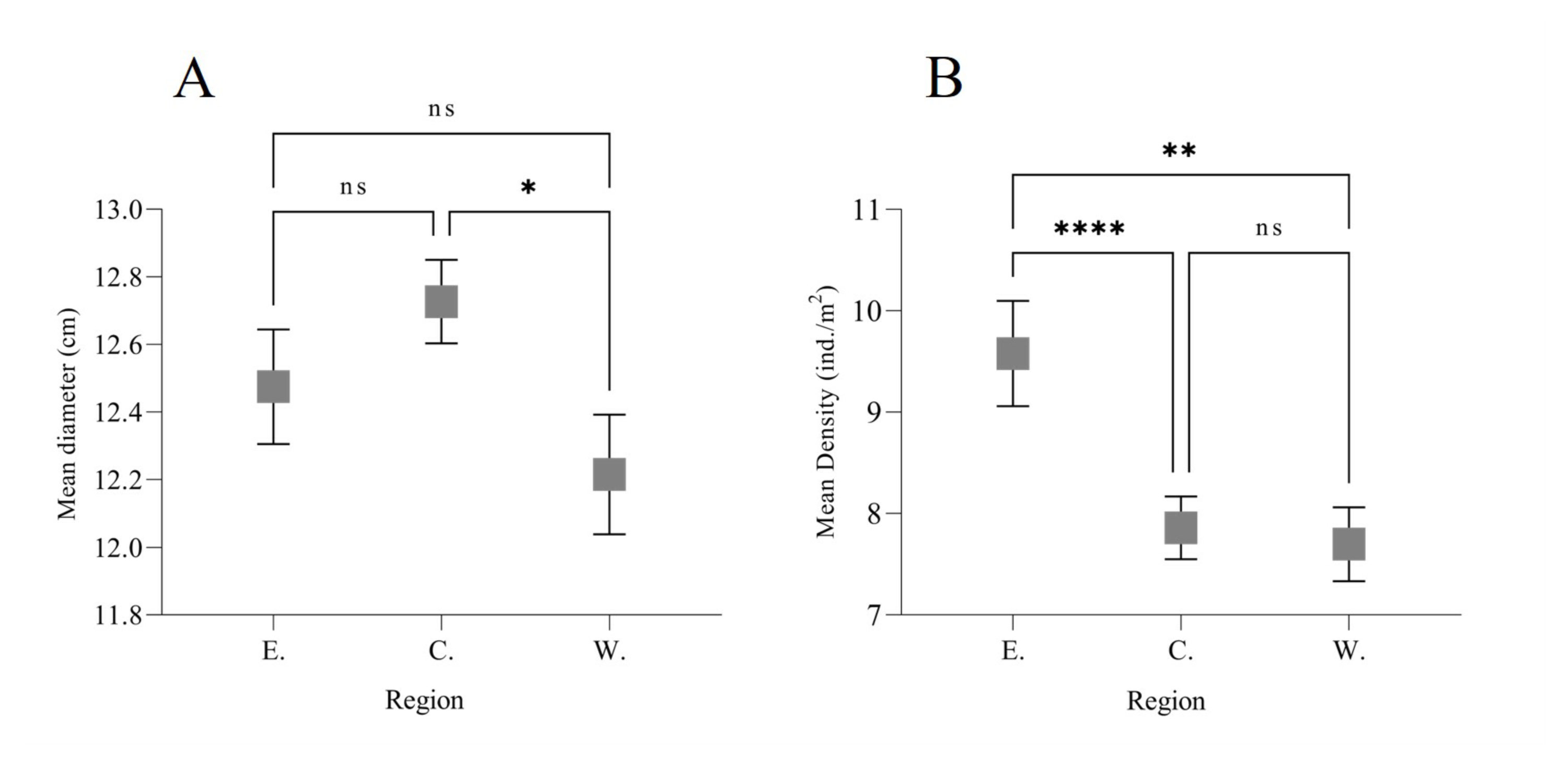

Supplement: Supplemental Information 1 — Each value is the mean ± SEM (N = 1400 quadrats). Kruskal-Wallis, H=6.212; p = 0.0448; Tukey, p < 0.05 (A) and Kruskal-Wallis, H=26.50; p < 0.0001; Tukey, p < 0.05 (B). [file peerj-11-15254-s001.png]
