# Supplementary material for: Characterization of the populations of upside-down jellyfish in Jardines de la Reina National Park, Cuba
Source: PeerJ. 2023 Apr 25;11:e15254. doi: 10.7717/peerj.15254 (PMC10143600; doi:10.7717/peerj.15254)
Supplement: Table S1 [file peerj-11-15254-s002.docx]

| **Region** | **Zone** | **Site** | **Latitude** | **Longitude** |
| --- | --- | --- | --- | --- |
| East | 1 | Mexicana | 20°45'55.9"N | 78°49'45.6"W |
|  | 2 | Peralta | 20°37'45.1"N | 78°33'32.4"W |
|  |  | Cayo Juan Grin | 20°38'28.9"N | 78°32'57.6"W |
|  | 3 | Cachiboca | 20°45'09.2"N | 78°49'12.8"W |
| Central | 4 | Cayo Piedra Piloto | 20°35'41.0"N | 78°27'13.0"W |
|  |  | Boca de las Anclitas | 20°47'32.8"N | 78°56'23.0"W |
|  |  | Laguna de las Anclitas | 20°47'41.3"N | 78°55'33.0"W |
|  |  | Laguna de las Anclitas Noroeste | 20°49'26.0"N | 78°56'05.6"W |
|  | 5 | Canal de las Auras | 20°53'42.1"N | 79°04'22.3"W |
|  |  | Estero de las Guasas Este | 20°49'43.4"N | 78°56'05.5"W |
|  |  | Canal de Caballones | 20°49'42.6"N | 78°55'47.8"W |
| West | 6 | Punta Oeste de Boca Grande | 21°00'23.5"N | 79°13'35.9"W |
|  | 7 | Cayo Alcatraz | 21°02'53.9"N | 79°17'58.3"W |
|  | 8 | Laguna de Bretón | 21°06'59.1"N | 79°26'34.7"W |
