# Supplementary material for: Characterization of the populations of upside-down jellyfish in Jardines de la Reina National Park, Cuba
Source: PeerJ. 2023 Apr 25;11:e15254. doi: 10.7717/peerj.15254 (PMC10143600; doi:10.7717/peerj.15254)
Supplement: Table S2 [file peerj-11-15254-s003.docx]

| **Sitio** | **Benthic Cover (%)** | **Reference** |
| --- | --- | --- |
| Cachiboca, PNJR,Cuba | 6.84 | This study |
| Canal de las Auras, PNJR,Cuba | 13.08 | This study |
| Estero de las Guasas, PNJR,Cuba | 23.22 | This study |
| Boca de las Anclitas, PNJR,Cuba | 23.72 | This study |
| Peralta, PNJR,Cuba | 19.63 | This study |
| Cayo Piedra Piloto, PNJR,Cuba | 13.79 | This study |
| Estero de las Guasas Este, PNJR,Cuba | 14.84 | This study |
| Caballones, PNJR,Cuba | 22.33 | This study |
| Cayo Juan Grin, PNJR,Cuba | 20.32 | This study |
| Laguna de las Anclitas, PNJR,Cuba | 10.98 | This study |
| Mexicana, PNJR,Cuba | 17.63 | This study |
| Punta Oeste de Boca Grande | 11.93 | This study |
| Cayo Alcatraz | 13.95 | This study |
| Laguna de Bretón | 15.35 | This study |
| Codrington, Barbuda | 17.8 | Zarnoch et al. (2020) |
| Aqaba, Jordania | 13.10 | Niggl & Wild (2010) |
| Isla Abaco, Bahamas | 12.40 | Stoner et al. (2011) |
| Isla Abaco, Bahamas | 7 – 10 | Stoner, Yeager & Layman (2014a); Stoner et al. (2014b) |
